# Identification and Quantification of Bioactive Molecules Inhibiting Pro-inflammatory Cytokine Production in Spent Coffee Grounds Using Metabolomics Analyses

**DOI:** 10.3389/fphar.2020.00229

**Published:** 2020-03-06

**Authors:** Khanh-Van Ho, Kathy L. Schreiber, Jihyun Park, Phuc H. Vo, Zhentian Lei, Lloyd W. Sumner, Charles R. Brown, Chung-Ho Lin

**Affiliations:** ^1^Center for Agroforestry, School of Natural Resources, University of Missouri, Columbia, MO, United States; ^2^Department of Food Technology, Can Tho University, Can Tho, Vietnam; ^3^Cell and Immunobiology Core, University of Missouri, Columbia, MO, United States; ^4^Metabolomics Center, University of Missouri, Columbia, MO, United States; ^5^Department of Biochemistry, Bond Life Sciences Center, University of Missouri, Columbia, MO, United States; ^6^Department of Veterinary Pathobiology, University of Missouri, Columbia, MO, United States

**Keywords:** untargeted analysis, metabolomics, phenolics, anti-inflammatory, spent coffee grounds

## Abstract

In this study, we assessed the anti-inflammatory properties of spent coffee grounds. Methanolic extracts of spent coffee grounds obtained from 3 Arabica cultivars possess compounds that exerted inhibitory effects on the secretion of inflammatory mediators (TNF-α, IL-6, and IL-10) induced by a human pro-monocytic cell line differentiated with PMA and stimulated with lipopolysaccharide (LPS). Our results indicated that the cytokine suppressive activities of the spent coffee ground (SCG) extracts were different among coffee cultivars tested. Hawaiian Kona extracts exhibited inhibitory effects on the expression of 3 examined cytokines, Ethiopian Yirgacheffe extracts reduced the secretion of TNF-α and IL-6, and Costa Rican Tarrazu extracts decreased the secretion of IL-6 only. Untargeted metabolomics analyses of SCG extracts led to the putative identification of 26 metabolites with known anti-inflammatory activities. Multiple metabolites (i.e., chrysin, daidzein, eugenol, naringenin, naringin, oxyresveratrol, pectolinarin, resveratrol, tectochrysin, theaflavin, vanillic acid, and vitexin rhamnoside) identified in the SCGs represent possible novel anti-inflammatory compounds. Of the 26 identified metabolites, the 12 compounds that had high relative intensities in all of the extracts were successfully quantified using liquid chromatography-tandem mass spectrometry analyses. Results from the targeted analyses indicated that caffeine and 5-caffeoylquinic acid (CQA) were the most abundant compounds in the SCG extracts. The contents of caffeine ranged from 0.38 mg/g (Ethiopian Yirgacheffe) – 0.44 mg/g (Costa Rican Tarrazu), whereas 5-CQA concentrations were in the range of 0.24 mg/g (Costa Rican Tarrazu) – 0.34 mg/g (Ethiopian Yirgacheffe). The presence of multiple anti-inflammatory compounds in SCGs provides a promising natural source for cosmetic and pharmaceutical industries.

## Introduction

Coffee, one of the most frequently consumed beverages, is the second greatest valuable commodity worldwide after petroleum (Murthy and Naidu, 2012). Approximately 6 million tons of spent coffee grounds (SCG), consisting of the solid residues produced during the brewing process, are produced annually on a global level (Getachew and Chun, 2017). The SCGs contain a rich source of amino acids, alkaloids, fatty acids, oils, polyphenols, minerals, and polysaccharides (Campos-Vega et al., 2015) and have been investigated for use as value-added products. The SCGs have been proposed to have a wide range of cosmetics application stemming from its phytochemical composition (e.g., phenolic acids, flavonoids, caffeine) (Campos-Vega et al., 2015). Other possible applications include fertilizers, absorbers, fillers and additives for polymer composites, supplements in animal feed, and biofuels (Givens and Barber, 1986; Castro et al., 2011; Park et al., 2016; Zarrinbakhsh et al., 2016; Moustafa et al., 2017; Ribeiro et al., 2017).

Coffee grounds were utilized as a folk medicine to treat dysentery, external wounds and inflammation (Hänsel et al., 1993). Native people also traditionally valued roasted coffee beans for medical purposes to treat anemia, edema, headaches, hepatitis, malaria, neuralgia, sleep disorders, and conditions of weakness (Hänsel et al., 1993; Rätsch, 2005). The SCGs are thought to exert multiple potential health benefits, but few studies have investigated the anti-inflammatory potential of these coffee residues. Ramalakshmi et al. (2009) reported no inhibitory effects of SCGs on expression of tumor necrosis factor (TNF)-α in the J774A.1 cell model system. In another study, López-Barrera et al. (2016) evaluated the effects of SCG fractions fermented by human gut flora on the cytokine secretion induced by lipopolysaccharide (LPS)-stimulated RAW 264.7 macrophages. This group reported that these SCG fractions significantly reduced the secretion of 3 cytokines [interleukin (IL)-1β, IL-10, and chemokine ligand (CCL) 17] out of the 40 examined cytokines/chemokines tested. The cytokine inhibition of the human gut fermented, unabsorbed SCG fractions were reported to be mediated primarily by short-chain fatty acids derived from dietary fiber (López-Barrera et al., 2016). In fact, multiple bioactive compounds in the SCGs (alkaloids and polyphenols) with known anti-inflammatory activities are potential sources of these cytokine suppressive effects. Similarly, bioactive compounds (e.g., caffeine, gallic acid, monocaffeoylquinic acids) identified in SCG have been reported to possess anti-inflammatory activities (Kim et al., 2005; Bravo et al., 2012; Hwang et al., 2014; Köroğlu et al., 2014). Despite these findings, the profiles of anti-inflammatory compounds in SCG from different coffee cultivars have not been adequately compared and characterized.

In this study, we described the inhibitory effects of SCG extracts derived from 3 Arabica cultivars on the expression of 3 inflammatory mediators (TNF-α, IL-6, and IL-10) using the human pro-monocytic cell line U-937. We subsequently identified and quantified the bioactive compounds in SCGs by metabolomics approaches. The integration of the cellular bioassay, untargeted chemical profiling, and targeted analyses is a powerful way to explore global activity metabolites with known as well as unknown physico-chemical properties (Rinschen et al., 2019).

## Materials and Methods

### Sample Preparation

Ground roast coffees from three Arabica cultivars including Costa Rican Tarrazu, Ethiopian Yirgacheffe, and Hawiian Kona were purchased from Lakota Coffee Company (Columbia, MO, United States). Spent coffee grounds from these coffee cultivars were obtained from a coffee maker (Bunn VP17-2, Springfield, IL, United States) after brewing of the ground roast coffee for 5 min at 90°C. The SCG was immediately homogenized using a coffee grinder (CBG100S, Black + Decker, Beachwood, OH, United States). The homogenized samples (25 g wet weight, 78% water) were extracted in 100 mL of methanol (HPLC grade, Fisher Scientific, Pittsburg, PA) twice and then sonicated at 10°C for 60 min as previously described in Ho et al. (2019). Subsequently, the methanolic extract was filtered through a 125 mm Whatman filter paper (GE Healthcare, Chicago, IL, United States) under SPE Vacuum Manifold (Visiprep^^TM^ SPE Vacuum Manifold, Sigma-Aldrich, United States), and then the supernatant was collected and stored at -20°C until analysis. The extract was allowed to thaw at room temperature, vortexed for 30 s, and then filtered through a 0.2μm syringe Anotop membrane filter (Whatman) prior to the analysis. For cellular assays, the resulting supernatant was evaporated until dryness under a flow of nitrogen. The dry extract was then resuspended with DMSO (Sigma-Aldrich, United States) at concentration of 5,000 mg/mL. Cytokine modulating activities of the extract were identified using BD^TM^ cytometric bead array (CBA) kits (BD Biosciences, San Jose, CA, United States). For untargeted chemical profiling and targeted analyses, the extracts from each cultivar were injected in triplicate into ultra-high performance liquid chromatography coupled with high resolution mass spectrometer (UHPLC-HRMS) and HPLC with tandem mass-spectrometer (HPLC-MS/MS), respectively.

### Cell Culture and Differentiation Induction

The human monocyte cell line U-937 was obtained from American Type Culture Collection (ATCC) (CRL-1593.2, ATCC, Manassas, VA, United States). Cells were grown in complete Roswell Park Memorial Institute (RPMI) medium (RPMI 1640, ATCC) supplemented with 10% fetal bovine serum (FBS, Sigma-Aldrich) and 100 μg/mL gentamicin at 37°C in a humidified incubator with 5% CO_2_. To induce differentiation of U-937 cells into macrophage-like cells, U-937 cells were seeded at 2 × 10^5^ cells/well in 96 well plates containing 50 nM of phorbol 12-myristate 13-acetate (PMA, Sigma-Aldrich) for 48 h (Rovera et al., 1979). Subsequently, the PMA-differentiated cells were washed twice and cultured in fresh growth media. The cells were incubated for an additional 18 h before pre-treatment with SCG extracts prepared from three cultivars. The SCG extracts were added in triplicate at 3 final concentrations (0.05, 0.5, and 5 mg/mL) for 2 h prior to stimulation with 1 μg/mL LPS (*Escherichia coli* 0127:B8, Sigma-Aldrich). Dexamethasone (Sigma-Aldrich), an anti-inflammatory agent known to inhibit cytokine secretion, was used as positive control at a concentration of 0.002 mg/mL. SCG extracts were resuspended in tissue culture-grade DMSO, and the highest concentration of DMSO in any sample was 0.1%. Therefore, a vehicle control of 0.1% DMSO was included in all experiments and served as a point of reference. After the addition of LPS for 22 h, the culture supernatants from each triplicate group were pooled, spun to remove cell debris, transferred to new tubes, and stored at -20°C until analysis.

### Cell Viability Analysis

MTT assays were performed to evaluate possible effects of the SCGs on cytotoxicity and/or cell loss. Briefly, cellular activity of mitochondrial dehydrogenase activity was measured using a colorimetric cell viability assay after removal of the supernatants. MTT substrate (Mosmann, 1983) was added to the cells in DMEM high glucose, phenol-red free media containing 1% FBS for 3 h at 37°C until formazan crystals were observed. Crystals were dissolved in acidified isopropanol, and samples were pipetted up and down several times to ensure that the crystals were completely dissolved before readings were taken. Absorbance was measured within 30 min after solvent addition using a BioTek ELx808 microplate reader (BioTek, Winooski, VT, United States). Formazan crystals were detected at a wavelength of 570 nm, and background absorbance was measured at 630 nm.

### Quantification of Cytokines/Chemokines Expression

A multiplex flow cytometric bead-based assay was used to quantitate the amount of secreted cytokines in the U-937 cells in the absence or presence of the SCG extracts. Multiple experiments were performed, and each cultivar was tested at 3 concentrations. Preparation of samples using the BD Cytometric Bead Array (CBA) human inflammatory cytokines kit was carried out according to the manufacturer’s recommendations. For these studies, the analysis focused on a subset of inflammatory cytokines (TNF-α, IL-6, and IL-10) present in the kit. Triplicate samples were collected on a BD LSR Fortessa X-20 cell analyzer (BD Biosciences, San Jose, CA, United States) using instrument settings suggested by BD and optimized in each experiment. A cytokine standard curve was included in each experiment, and cytokine levels were calculated from a five-parameter logistic curve using a curve-fitting software.

### Untargeted Metabolomics Analyses to Putatively Identify Anti-inflammatory Molecules

The extracts were analyzed using an UHPLC system coupled to a maXis impact quadrupole-time-of-flight high-resolution mass spectrometer (Q-TOF) (Bruker Co., Billerica, MA, United States) operated in both negative and positive electrospray ionization modes with the nebulization gas pressure at 43.5 psi, dry gas of 12 L/min, dry temperature of 250°C and a capillary voltage of 4000 V, as described in Ho et al. (2018). The SCG extracts obtained from 3 Arabica cultivars were separated using a Waters Acquity UHPLC BEH C18 column (2.1 × 150 mm, 1.7 μm particles size) at 60°C. The solvent system was 0.1% formic acid in water (A) and 100% acetonitrile (B). The gradient elution used started with a linear gradient of 95%: 5–30%: 70% eluents A: B in 30 min. Subsequently, the separation was followed by a linear wash gradient as follows 70–95% B, 95% B, 95–5% B, and 5% B at 30–33 min, 33–35 min, 35–36 min, and 37–40 min, respectively. The flow rate was 0.56 mL/min. Mass spectral data were collected automatically using a scan range from m/z 100 to 1,500 and auto-calibrated using sodium formate after data acquisition. Each coffee cultivar and methanol blank (served as a control) were analyzed in triplicate.

### Targeted Metabolomics Analysis to Determine Contents of Major Anti-inflammatory Molecules

Major metabolites that had high relative intensities across the SCG extracts were selected for absolute quantification of their contents using liquid chromatography-tandem mass spectrometry (LC-MS/MS) analysis with authentic standards. The LC-MS/MS analyses were performed using an HPLC system (Water Alliance 2695, Water Co., Milford, MA, United States) coupled to a Waters Acquity TQ triple quadrupole mass spectrometer operated in negative electrospray ionization mode with the nebulization gas pressure at 43.5 psi, dry gas of 12 L/min, dry temperature of 250°C and a capillary voltage of 1500 V. The compounds in the SCG extracts (20 μL volume per injection) from three coffee cultivars were separated using a Phenomenex Kinetex C18 reverse-phase column (100 × 4.6 mm; 2.6 μm particle size, Torrance, CA, United States) at 25°C. The mobile phases were 0.1% formic acid in water (A) and 100% acetonitrile (B). The gradient elution used were 2% B, 2–80% B, 80–98% B, 2% B at 0–0.5 min, 0.5–7 min, 7.0–9.0 min, 9.0–15.0 min, respectively at a flow rate of 0.5 mL/min. MS detection was performed by MS/MS using the multiple reaction monitoring (MRM) mode. Waters IntelliStart optimization software was used to optimize collision, ionization energy, MRM and SIR (single ion recording) transition ions (molecular and product ions), capillary and cone voltage, desolvation gas flow, and collision energy. Waters Empower 3 software was used to analyze data. The concentrations of major anti-inflammatory compounds found in SCG extracts were determined based on a standard curve for each analyte generated using authentic standards of these compounds (purity > 95%, Sigma-Aldrich) at 8 concentrations (0.01, 0.025, 0.05, 0.1, 0.5, 1, 5, 10 ppm) in triplicate.

Assessment of the sensitivity of the analytical method was performed by calculating the limit of detection (LOD) and limit of quantification (LOQ). The LOD and LOQ for each compound were calculated by employing signal-to-noise ratios of three and ten. The extraction recover rates of the fortified internal standards colchicine and β-naphthylsulfate were used to determine the efficiencies of the extraction procedure. For the extraction procedure, 100 μg of each internal standard was fortified. The recovery rates were greater than 95%.

### Data Processing and Statistical Analysis

Cell viability and cytokine concentrations were determined relative to control samples (in the presence of DSMO vehicle and without cultivars). Relative cell viability (%) was obtained by dividing the MTT absorbance of treated samples by those in control samples, and multiplying by 100. Relative cytokine secretions (%) were calculated by dividing the cytokine concentration of treated samples by those in control samples, and multiplying by 100. Relative cell viability, relative cytokine secretion, concentrations of anti-inflammatory compounds obtained from LC-MS/MS analysis were analyzed as a completely randomized design using PROC MIXED in SAS 9.4 (SAS Institute, Cary, NC, United States). The coffee extract (treatment) was the fixed effect and replication was the random variable. Differences between extracts were determined using Fisher’s LSD at *p* < 0.01.

The UHPLC-MS data was analyzed based on the procedure described by Ho et al. (2019). First, the original LC-MS data files were converted to NetCDF format files and were uploaded to XCMS online platform^1^ (Tautenhahn et al., 2012). The XCMS data were processed with parameters as follows: pairwise analyses between each coffee cultivar and the control (methanol) were conducted in centWave mode for feature detection (1 m/z = 10 ppm, minimum peak width = 5 s, and maximum peak width = 20 s), an obiwarp method was selected for retention time correction (profStep = 1), chromatogram alignment was set as minfrac = 0.5, bw = 5, mzwid = 0.015, max = 100, minsamp = 1, adducts was optimized for UPLC/Bruker Q-TOF in both ESI(+) and ESI(-) and plant was selected for sample biosource for identification, and the unpaired parametric *t*-test (Welch *t*-test) was used for the statistical analysis. Metabolites of significant features (*p* < 0.01 and intensity ≥ 10,000) were putatively identified based on the accurate mass of the molecular ions, referenced to METLIN metabolite mass spectral database containing over 1 million molecules^2^ (Guijas et al., 2018). Metabolites with known to possess anti-inflammatory activities were used to predict anti-inflammatory compounds in each cultivar (Supplementary Table S1). To further characterize differences in profiles of anti-inflammatory compounds among examined coffee cultivars, partial least squares-discriminant analysis (PLS-DA) was performed via a web-based tool MetaboAnalyst (Chong et al., 2018).

## Results

### Cell Viability Analysis

Cell viability assays were performed to address any potential cytotoxic effects of the SCG cultivars. A reduction in MTT absorbance could result from a loss of cell viability, a reduction in cell number, a decrease in mitochondrial activity, an inhibition of cytokine secretion, or a combination of these factors. The MTT viability assays were carried out immediately after the cell supernatants were collected. Dexamethasone, a known inhibitor of cytokine secretion, was included as a positive control. Preliminary experiments using a wide range of DMSO concentrations indicated that DMSO did not affect U-937 cell number or viability as assessed by trypan blue exclusion (data not shown) at the concentrations used in this study, indicating that DMSO is not toxic under the experimental conditions. Therefore, a reduction in MTT absorbance in the presence of the cultivars would indicate a toxic effect of the cultivar rather than the vehicle.

**FIGURE 1:**
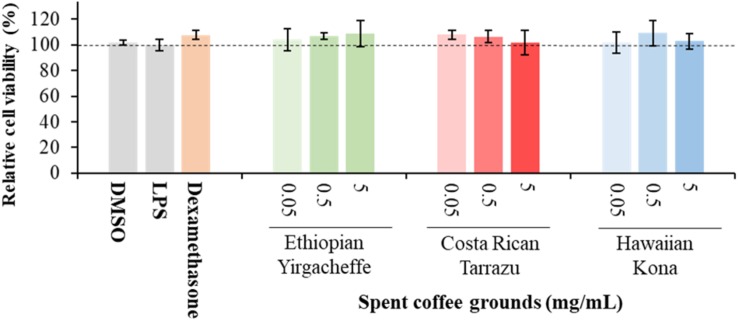
Effect of DMSO, lipopolysaccharide (LPS), Dexamethasone, and spent coffee ground extracts on the viability of PMA-differentiated U-937 cells. DMSO, PMA-differentiated cells treated with 0.1% DMSO, no LPS, no coffee extracts; LPS, LPS and DMSO; coffee extracts, coffee extracts, DMSO and LPS; Dexamethasone, 0.2 μg/mL dexamethasone, DMSO, and LPS. Mean ± SEM.

The results shown in Figure 1 illustrate that cell viability in all treatment groups (PMA-differentiated U-937 cells with the addition of SCGs, dexamethasone, LPS) was not significantly different compared to those in the control cultures (PMA-differentiated U-937 cells with DMSO but without LPS and SCG extracts). These results demonstrate that the SCG extracts and dexamethasone did not show any toxic effects on PMA-differentiated U-937 cells at any of the concentrations tested, and they were further examined for their effects on cytokine secretion.

### Impact of Spent Coffee Ground Extracts on Cytokine Secretion

Cytokine secretion was analyzed as a possible indicator of systemic inflammation using the U-937 model system. Secreted cytokine levels were measured in the absence or presence of SGC extracts, with the results expressed relative to control samples cultured with vehicle in the absence of SCG extracts. Three representative cytokines, TNF-α, IL-6, and IL-10, were chosen to reflect both anti-inflammatory as well as pro-inflammatory properties.

**FIGURE 2:**
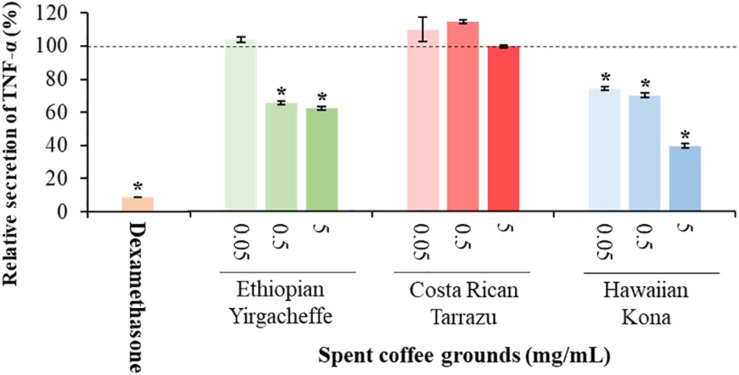
Effect of spent coffee ground extracts on the secretion of TNF-α by PMA-differentiated, LPS-stimulated U937 cells. Dexamethasone was used at 0.2 μg/mL as a positive control. (*) Significant decrease (*p* < 0.001) compared to PMA-differentiated, LPS-stimulated U937 cells in the absence of extract. Mean ± SEM.

**FIGURE 3:**
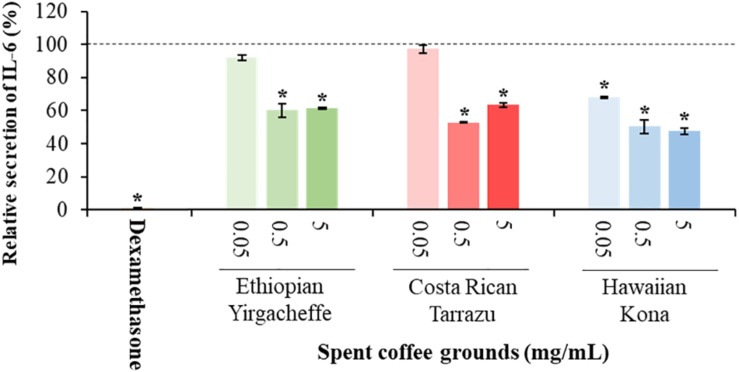
Effect of spent coffee ground extracts on the secretion of IL-6 by PMA-differentiated, LPS-stimulated U937 cells. Dexamethasone was used at 0.2 μg/mL as a positive control. (*) Significant decrease (*p* < 0.001) compared to PMA-differentiated, LPS-stimulated U937 cells in the absence of extract. Mean ± SEM.

**FIGURE 4:**
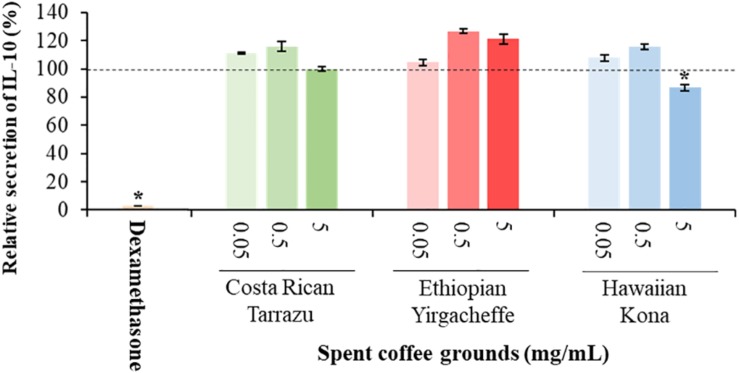
Effect of spent coffee ground extracts on the secretion of IL-10 by PMA-differentiated, LPS-stimulated U937 cells. Dexamethasone was used at 0.2 μg/mL as a positive control. (*) Significant decrease (*p* < 0.001) compared to PMA-differentiated, LPS-stimulated U937 cells in the absence of extract. Mean ± SEM.

As shown in Figures 2–4, inclusion of Hawaiian Kona extracts in the cultures for 2 h prior to the addition of LPS reduced the levels of all 3 cytokines. This effect was dose-dependent, illustrated by a reduction in TNF-α secretion of 25.8, 29.9, and 60.5%, and in IL-6 levels by 32.1, 49.7, and 52.5%, at concentrations of 0.05, 0.5, and 5.0 mg/mL, respectively. TNF-α levels were reduced by 34.4 and 37.8% after pre-incubation of SCG extracts obtained from Ethiopian Yirgacheffe at concentrations of 0.5 and 5.0 mg/mL, respectively, but were not statistically different following pre-incubation with Costa Rican Tarrazu SCGs. A similar reduction in IL-6 levels was observed in cultures containing Ethiopian Yirgacheffe and Costa Rican Tarrazu extracts at the two highest concentrations of 0.5 and 5.0 mg/mL, with a decrease of 40.0, and 38.9%, and of 47.2 and 36.6%, respectively. No effect on IL-6 levels was observed at the lower concentration of 0.05 mg/mL when U-937 cells were cultured with either extract. The effect on IL-10 secretion was minimal for all three SCG extracts, and the only minor effect noted was a 13.3% reduction after addition of the Hawaiian Kona extract at 5.0 mg/mL. Cytokine levels were reduced to over 90% for all 3 cytokines examined in the presence of the positive control agent dexamethasone, a known inhibitor of cytokine secretion.

### UHPLC-HRMS Analyses Revealed Anti-inflammatory Metabolite Profiles of the Spent Coffee Grounds

**FIGURE 5:**
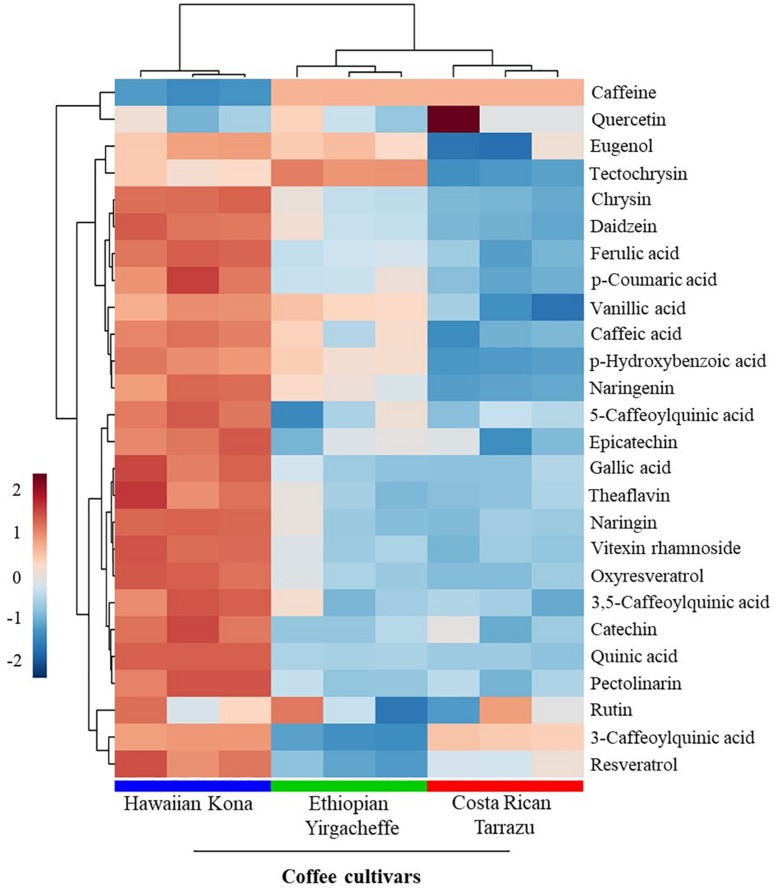
Relative intensities of metabolites with known anti-inflammatory activities in spent coffee grounds. In the heatmap, red represents higher relative abundance, whereas blue represents lower relative abundance.

LC-MS fingerprints of anti-inflammatory molecules in the extracts were obtained from the liquid chromatography–high resolution MS (LC-HRMS) analysis. The UHPLC-HRMS data processed with XCMS Online resulted in 2,418 significant features in ionization positive mode and 162 features in the negative ionization mode. These features were annotated using METLIN metabolite database, which resulted in the putative identification of 26 metabolites with known anti-inflammatory activities (Supplementary Table S1). These metabolites included a alkaloid and polyphenolic compounds (Table 1). Twelve out of 26 anti-inflammatory metabolites that had high relative intensities across the SCG extracts of all coffee cultivars (Figure 5) were further selected for the absolute quantification of the concentrations in the SCG extracts by LC-MS/MS analyses with authentic standards (Tables 2, 3).

**TABLE 1:** Metabolites with known anti-inflammatory activities in spent coffee grounds from three Arabica cultivars via untargeted metabolomics analyses.

**No.**	**Putatively identified compound**	**Retention time (min)**	**Theoretical mass**	**Exact mass**	**Δppm**	**Adducts**	**Predicted formula**
1	3-Caffeoylquinic acid^a,b^	1.56	353.0866	353.0872	–1.70	[M-H]	C_16_H_18_O_9_
2	5-Caffeoylquinic acid^a,b^	2.24	353.0871	353.0872	–0.28	[M-H]	C_16_H_18_O_9_
3	3,5-Caffeoylquinic acid^a^	6.67	539.1162	539.1165	–0.56	[M + Na]	C_25_H_24_O_12_
4	Caffeic acid^a,b^	1.55	179.0348	179.0344	2.23	[M-H]	C_9_H_8_O_4_
5	Caffeine^a,b^	0.41	195.0881	195.0882	–0.51	[M + H]	C_8_H_10_N_4_O_2_
6	Catechin^a,b^	3.74	289.0697	289.0712	–5.19	[M-H]	C_15_H_14_O_6_
7	Chrysin^a^	20.28	255.0655	255.0657	–0.78	[M + H]	C_15_H_10_O_5_
8	Daidzein^a^	20.28	255.0655	255.0657	–0.78	[M + H]	C_15_H_10_O_4_
9	Epicatechin^a,b^	2.68	289.0708	289.0712	–1.38	[M-H]	C_15_H_14_O_6_
10	Eugenol^a^	5.55	165.0926	165.0916	6.06	[M + H]	C_10_H_12_O_2_
11	Ferulic acid^a,b^	2.67	193.0504	193.0501	1.55	[M-H]	C_10_H_10_O_4_
12	Gallic acid^a,b^	16.05	208.9846	208.9852	–2.87	[M + K]	C_7_H_6_O_5_
13	Naringenin^a^	5.29	271.0610	271.0606	1.48	[M-H]	C_15_H_12_O_5_
14	Naringin^a^	19.21	581.1843	581.1870	–4.65	[M + H]	C_27_H_32_O_14_
15	Oxyresveratrol^a^	13.65	407.1341	407.1342	–0.25	[M + H]	C_20_H_22_O_9_
16	p-Coumaric acid^a,b^	2.17	163.0403	163.0395	4.91	[M-H]	C_9_H_8_O_3_
17	p-Hydroxybenzoic acid^a,b^	0.76	139.0386	139.0395	–6.47	[M-H]	C_7_H_6_O_3_
18	Pectolinarin^a^	31.87	621.1827	621.1819	1.29	[M-H]	C_29_H_34_O_15_
19	Quercetin^a^	6.73	489.1359	489.1397	–7.77	[M-H]	C_24_H_26_O_11_
20	Quinic acid^a,b^	0.59	191.0562	191.0556	3.14	[M-H]	C_7_H_12_O_6_
21	Resveratrol^a^	7.71	229.0862	229.0864	–0.87	[M + H]	C_14_H_12_O_3_
22	Rutin^a^	13.65	407.1341	407.1342	–0.25	[M + H]	C_20_H_22_O_9_
23	Tectochrysin^a^	8.42	291.0627	291.0633	–2.06	[M + Na]	C_16_H_12_O_4_
24	Theaflavin^a^	13.01	565.1354	565.1346	1.42	[M + H]	C_29_H_24_O_12_
25	Vanillic acid^a,b^	3.70	335.0758	335.0767	–2.69	[M + H]	C_16_H_14_O_8_
26	Vitexin rhamnoside^a^	15.20	601.1539	601.1533	1.00	[M + Na]	C_27_H_30_O_14_

**^*a*^Putative identification by UHPLC-HRMS. ^*b*^Absolute concentrations were determined by LC-MS/MS with authentic standards.**

**TABLE 2:** Molecular and product ions, retention times, linear correlation coefficients, LOD, and LOQ of anti-inflammatory compounds identified in spent coffee ground extracts.

**Compound**	**Molecular ions (m/z)**	**Product ions (m/z)**	**Retention time (min)**	**Polarity**	**Linear equation^a^**	**Correlation coefficient (*R*^2^)**	**LOD^*b*^ (μg/g)**	**LOQ^*c*^ (μg/g)**
5-Caffeoylquinic acid	353	191.04	5.71	ES-	*y* = 96973x	0.9963	0.043	0.142
Quinic acid	190.83	85	2.17	ES-	*y* = 123139x	0.9985	0.023	0.076
Vanillic acid	166.86	–	6.75	ES-	*y* = 21505x	0.9978	0.104	0.346
Caffeic acid	178.88	135	6.65	ES-	*y* = 745425x	0.9994	0.010	0.033
Epicatechin	288.63	109	6.54	ES-	*y* = 22414x	0.9912	0.084	0.279
p-Hydroxybenzoic acid	136.88	93	6.52	ES-	*y* = 269043x	0.9929	0.030	0.100
Catechin	288.65	109.2	6.31	ES-	*y* = 18248x	1.00	0.056	0.186
Ferulic acid	193	134.09	7.43	ES-	*y* = 131247x	0.9993	0.021	0.069
p-Coumaric acid	163.07	119	7.26	ES-	*y* = 403050x	0.9914	0.012	0.04
3-Caffeoylquinic acid	353	191	2.51	ES-	*y* = 541946x	0.9995	0.005	0.017
Gallic acid	169	125	2.48	ES-	*y* = 234571x	0.9862	0.005	0.016
Caffeine	195.01	137.9	6.28	ES+	*y* = 2E-07x	0.9842	0.002	0.007

**^*a*^Linear equation indicate relationship between peak area of anti-inflammatory compounds and concentration in standard solutions. ^*b*^LOD, limit of detection.^*c*^LOQ, limit of quantitation.**

**TABLE 3:** Polyphenols and caffeine (LC-MS/MS) contents (μg/g of dry weight) in spent coffee grounds.

**Polyphenols**	**Coffee cultivars**		
	Ethiopian Yirgacheffe*	**Costa Rican Tarrazu**	**Hawaiian Kona**
5-Caffeoylquinic acid	338.1 ± 1.7^a^	236.7 ± 1.5^*c*^	293.5 ± 1.8^*b*^
Quinic acid	207.4 ± 0.5^*c*^	238.3 ± 0.5^a^	218.8 ± 0.3^*b*^
Vanillic acid	86.2 ± 2.9^a^	53.9 ± 1.3^*b*^	54.3 ± 1.4^*b*^
Caffeic acid	54.5 ± 1.5^a^	41.4 ± 0.9^*b*^	43.4 ± 0.5^*b*^
Epicatechin	37.2 ± 1.5	<LOD**	<LOD
p-Hydroxybenzoic acid	27.9 ± 0.3^a^	1.9 ± 0.2^*b*^	2.2 ± 0.1^*b*^
Catechin	24.0 ± 2.2^a^	1.9 ± 0.3^*b*^	1.4 ± 0.3^*b*^
Ferulic acid	21.1 ± 0.6	<LOD	<LOD
p-Coumaric acid	18.3 ± 1.8^a^	0.7 ± 0.1^*b*^	0.7 ± 0.1^*b*^
3-Caffeoylquinic acid	3.9 ± 0.5	2.8 ± 0.0	3.7 ± 0.0
Gallic acid	3.1 ± 0.0^a^	1.0 ± 0.0^*b*^	1.1 ± 0.0^*b*^
Total polyphenols	821.5 ± 5.7^a^	578.6 ± 1.7^*c*^	619.1 ± 0.7^*b*^
Caffeine	384.5 ± 1.1^*b*^	439.0 ± 4.0^a^	426.9 ± 1.3^a^

*All values are shown as mean ± SE (n = 3). In each row, different letter indicate significant differences (p < 0.01) among different spent coffee grounds.**LOD, limit of detection.

### LC-MS/MS Analyses Indicated Significant Differences in Anti-inflammatory Profiles of Three Coffee Cultivars

All 12 selected anti-inflammatory metabolites including caffeine, 5-caffeoylquinic acid (5-CQA), quinic acid, vanillic acid, caffeic acid, epicatechin, catechin, ferulic acid, 3-CQA, p-coumaric acid, p-hydroxybenzoic acid, gallic acid were found in Ethiopian Yirgacheffe extracts, while 10 out of 12 metabolites were detected in Costa Rican Tarrazu and Hawaiian Kona extracts (Table 3). The contents of these anti-inflammatory compounds in SCG vary among tested coffee cultivars. Caffeine, a methylxanthine alkaloid, was the most dominant compound in all examined coffee cultivars. The concentrations of caffeine were found to be the significant higher in Costa Rican Tarrazu (439.0 ± 4.0 μg/g) and Hawaiian Kona (426.9 ± 1.3 μg/g) than that in Ehiopian Yirgacheffe (384.5 ± 1.1 μg/g). The most abundant phenolic compound in all examined coffee cultivars was 5-CQA. Ehiopian Yirgacheffe contained the richest abundance of 5-CQA (338.1 ± 1.7 μg/g), followed by Hawaiian Kona (293.5 ± 1.8 μg/g) and Costa Rican Tarrazu (236.7 ± 1.5 μg/g). The abundance of quinic acid ranked second among the examined phenolic compounds. This compound was found to be at the highest amount in Costa Rican Tarrazu (238.3 ± 0.5 μg/g), followed by and Hawaiian Kona (218.8 ± 0.3 μg/g) and Costa Rican Tarrazu (207.4 ± 0.5 μg/g). The contents of other compounds with an exception of 3-CQA were found to be present at the higher abundances in Ehiopian Yirgacheffe compared to other cultivars, while there were no significant differences in the contents of these compounds in Costa Rican Tarrazu and Hawaiian Kona. The concentrations of vanillic acid, caffeic acid, catechin, p-coumaric acid, p-hydroxybenozic acid in Ehiopian Yirgacheffe were 86.2 ± 2.8 μg/g, 54.5 ± 1.5 μg/g, 24.0 ± 2.2 μg/g, 18.3 ± 1.8 μg/g, 27.9 ± 0.3 μg/g, respectively, while that of Costa Rican Tarrazu and Hawaiian Kona were 53.9 ± 1.3 μg/g and 54.3 ± 1.4 μg/g, 41.5 ± 0.9 μg/g and 43.4 ± 0.5 μg/g, 1.9 ± 0.3 μg/g, and 1.4 ± 0.3 μg/g, 0.7 ± 0.1 μg/g and 0.7 ± 0.1 μg/g, 1.9 ± 0.2 μg/g, and 2.2 ± 0.1 μg/g, respectively. Epicatechin and ferulic acid were not detectable in Costa Rican Tarrazu and Hawaiian Kona extracts, but were present in Ehiopian Yirgacheffe extracts at concentrations of 37.2 ± 1.5 μg/g and 21.1 ± 0.6 μg/g, respectively. No significant difference in the abundance of 3-CQA was found among all SCG extracts. This compound was found to be at minor levels in all three coffee extracts with its contents ranging from 2.9 ± 0.2 μg/g (Costa Rican Tarrazu) to 3.9 ± 0.5 μg/g (Hawaiian Kona).

**FIGURE 6:**
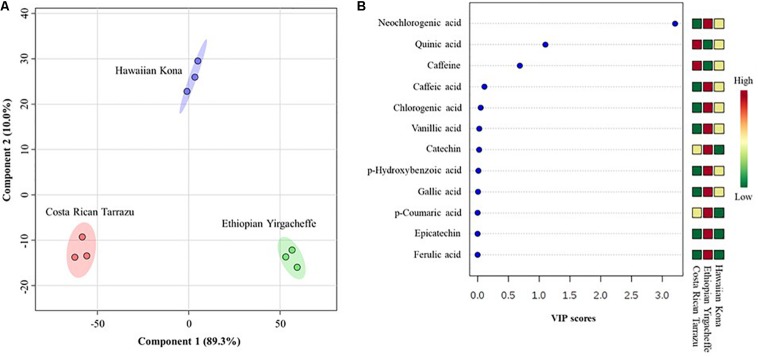
Differences in anti-inflammatory profiles of spent coffee grounds from three coffee cultivars. **(A)** Partial least squares-discriminant analysis (PLS-DA), **(B)** Variable importance in projection (VIP). In the PLS-DA plot, circles with same colors represent replicates of metabolic profiles for each cultivar. The colored ellipses indicate 95% confidence regions of anti-inflammatory profiles for each cultivar. In the VIP score plot, the colored boxes on the right indicate the relative abundance of the corresponding anti-inflammatory compounds in each cultivar. Red represents higher relative abundance, whereas green and blue represent lower relative abundance in the VIP score plot.

To further characterize differences in anti-inflammatory profiles in SCGs obtained from different coffee cultivars, partial least squares-discriminant analysis (PLS-DA) were performed. A cross-validation method was utilized to evaluate model quality and resulting R2 and Q2 values were 0.99 and 0.97, respectively, indicating that the model was reliable. PLS-DA score plot with two principal components covered 99.3% of total variability of the data (Figure 6A), revealing significant differences in anti-inflammatory profiles in SCG derived from different examined coffee cultivars. The first principal components (PC1) explained 89.3% of the total variability of the data, whereas the second principal components (PC2) accounted for 10.0% of the total variability of the data set. In the PLS-DA score plot, all 3 coffee cultivars were distributed separately along the PC1. Regarding the PC2, Costa Rican Tarrazu and Ethiopian Yirgacheffee relatively shared a similar pattern, and Hawaiian Kona differed from other cultivars. Variable importance in projection (VIP) analyses revealed 5-CQA, quinic acid, and caffeine were the most important compounds (Figure 6B). These compounds also were major compounds found in all tested cultivars (Table 3).

## Discussion

Given the huge availability of SCGs, determination of bioactive compounds found within complex mixtures is an important step toward developing novel value-added byproducts that may potentially increase the sustainability of the coffee agro-industry (Kovalcik et al., 2018). In the present study, we demonstrated that methanolic extracts of SCGs possess compounds that exerted inhibitory effects on the secretion of inflammatory mediators (TNF-α, IL-6, and IL-10) induced in a human pro-monocytic cell line differentiated with PMS and stimulated with LPS. The pro-inflammatory mediators TNF-a and IL-6 are key regulators of innate and adaptive immune responses, and they play a role in disease onset and persistence. As such, they offer potential therapeutic targets for the treatment of acute chronic diseases such as rheumatoid arthritis (Smolen and Aletaha, 2015). IL-10 is a potent immune-modulatory cytokine that has broad anti-inflammatory properties (O’Garra et al., 2008), including the inhibition of TNF production (Smallie et al., 2010). Our results indicated that the cytokine suppressive activities of SCG extracts were different among the coffee cultivars tested. Hawaiian Kona extracts affected the secretion of all 3 examined cytokines in the U-937 model system, whereas Ethiopian Yirgacheffe extracts reduced the secretions of TNF-α and IL-6 only and Costa Rican Tarrazu decreased the secretion of IL-6 only. Cell viability is similar in the absence and presence of all three SCGs extracts, demonstrating that the reduction in cytokine levels is not a result of direct toxic effects. It is possible that the varying levels of cytokine suppressive activities among the Arabica cultivars arise from the differences in the composition and proportions of the anti-inflammatory compounds present in the different SCG extracts (Figure 6). The presence of multiple compounds in the cultivars also raises the possibility that different compounds in the same mixture may modulate each other’s activity. For example, the overall cytokine concentration may be determined by a net balance between stimulatory and inhibitory activities. Likewise, there might be synergism between two inhibitory compounds, resulting in a more profound decrease in cytokine release. The involvement of multiple compounds on cytokine secretion might explain why Hawaiian Kona extracts exhibited inhibitory effects on all three cytokines, whereas the other cultivars had a more limited effect on cytokine secretion. Our findings suggested that SCG extracts from Hawaiian Kona have very distinct profiles of anti-inflammatory molecules and other metabolites as compared to the extracts from other cultivars (Figures 5, 6). The significant higher levels of the bioactive compounds with known anti-inflammatory activities in the Hawaiian Kona extracts (Figure 5), such as caffeine, 5-caffeoylquinic acid, and quinic acid might be directly or synergistically responsible for the inhibitory activities (Table 3). Furthermore, other materials (e.g., polysaccharides, ash, minerals) found in SCGs (McNutt and Quan, 2019) could be exerting an effect on IL-10 secretion in the U-937 model system. Cytokines are soluble factors that play a role in various steps of acute and chronic inflammation (Brenner et al., 2014). Taken together, our findings suggest that SCGs present a promising source of anti-inflammatory mediators for use in the pharmaceutical and cosmetic industries.

So far, only few studies have examined potential anti-inflammatory activities of SCGs in mouse cell line model systems. Ramalakshmi et al. (2009) evaluated SCG extracts derived from Arabia plantation and Robusta cherry, two varieties of graded coffee beans, on the expression of TNF-α in J774A.1 mouse cell line. Their results showed that TNF-α levels were not suppressed following addition of these two extracts at the 3 concentrations tested (1, 3, and 10 μg/mL). Our results demonstrated inhibitory activity on TNF-α secretion in the human U-937 model system after pre-incubation with Ethiopian Yirgacheffe and Hawaiian Kona extracts. However, the effect was observed in our study using higher concentrations (500 and 5,000 μg/mL) of SCGs compared to those used in the Ramalakshmi et al. study. López-Barrera et al. (2016) evaluated the effects of SCG fractions fermented by human gut flora on the cytokine secretion in mouse RAW 264.7 macrophages stimulated with LPS. This group reported that out of the 40 cytokines/chemokines measured, the level of only 3 cytokines (IL-1β, IL-10, and CCL17) was significantly reduced. The cytokine inhibitory activity of the gut fermented, unabsorbed SCG fractions was mainly mediated by short-chain fatty acids derived from dietary fiber (López-Barrera et al., 2016). Our results are consistent with other studies showing an effect of roasted coffee bean extracts on cytokine expression. Jung et al. (2017) investigated the role of different roasting levels (Light, Medium, City, and French roast) on the secretion of TNF-α and IL-6 in RAW 264.7 mouse cell line. There findings showed that mRNA expression of TNF-α and IL-6 was reduced in the LPS-stimulated RAW 264.7 cells relative to control cells. In addition, the expression of TNF-α and IL-6 was decreased more as the roasting levels increased. An amount of inhibition comparable to that reported in our study was observed when the light roast coffee extract was tested at the highest concentration, 2 mg/mL, resulting in 42.9% and 36.7% inhibition of TNF-α and IL-6, respectively. The magnitude of this inhibition is comparable to that observed when Hawaiian Kona extracts were tested at 5 mg/mL, whereby a 60.5 and 52.5% reduction was found in TNF-α and IL-6 secreted protein levels, respectively.

Our results demonstrated a diverse range of the anti-inflammatory bioactive compounds in SCGs. In fact, 26 anti-inflammatory metabolites in the SCG extracts were putatively identified via untargeted metabolomics analyses and 12 anti-inflammatory compounds were successfully confirmed and quantified in SCG extracts by LC-MS/MS analyses with authentic reference standards (Tables 1–3). Among 26 anti-inflammatory metabolites, 12 compounds including chrysin, daidzein, eugenol, naringenin, naringin, oxyresveratrol, pectolinarin, resveratrol, tectochrysin, theaflavin, vanillic acid, and vitexin rhamnoside were the first report possibly present in SCGs, whereas other compounds have been documented as polyphenolic compounds in SCGs (Andrade et al., 2012; López-Barrera et al., 2016). Future research will focus on purification and characterization of compounds mainly driving the cytokine suppressive activities in SCGs.

Among the identified anti-inflammatory compounds, our results revealed that caffeine and 5-caffeoylquinic acid (5-CQA), a monocaffeoylquinic acid (CQA), were the most abundant compounds in the SCG extracts from all examined coffee cultivars. The contents of caffeine ranged from 0.38 mg/g (Ethiopian Yirgacheffe) – 0.44 mg/g (Costa Rican Tarrazu), whereas 5-CQA concentrations were in the range of 0.24 mg/g (Costa Rican Tarrazu) – 0.34 mg/g (Ethiopian Yirgacheffe). Caffeine is well-known as a signature compound in coffee and coffee byproducts. López-Barrera et al. (2016) reported that caffeine contents in Arabica SCG obtained from 2 roasted levels (medium and dark roasted) with Soxhlet extraction were approximately 0.4 mg/g, which roughly shared similar values of caffeine found in this study. However, Bravo et al. (2012) previously reported the contents of caffeine of Arabica SCG with Soxhlet extraction were in the range of 3.6–5.2 mg/g depending on the coffeemakers (filter, espresso, and plunger), which were > 9 times higher than the values observed in López-Barrera et al. (2016) and our study. Caffeoylquinic acids [monocaffeoylquinic and dicaffeoylquinic acids (diCQA)] have been documented as the most abundant phenolic compounds in spent coffee grounds (Campos-Vega et al., 2015), in which 5-CQA was the most abundant compound in SCGs obtained from both Arabica and Robusta varieties (Bravo et al., 2012). Bravo et al. (2012) reported that the contents of 5-CQA in Arabica spent coffee grounds obtained from different coffeemakers including filter, espresso, plunger were 3.6, 2.8, 2.5 mg/g respectively, which was 8–15 times higher than the values observed in our study.

Differences in the levels of bioactive compounds in SCG among different studies are likely due to the differences in coffee materials (coffee varieties, cultivars, geographic sources, growth conditions), roasting processes and extraction preparation. Our results indicated that the SCGs obtained from 3 different Arabica cultivars differed on the contents of anti-inflammatory compounds (Table 3). In fact, the total phenolic compounds of SCGs were found to be highest in Ethiopian Yirgacheffe (0.82 mg/g), followed by Hawaiian Kona (0.62 mg/g), and then Costa Rican Tarrazu (0.58 mg/g). The concentrations of bioactive compounds in SCGs have been previously reported to be variable among coffee varieties. Arabica spent coffee grounds contained less caffeine than Robusta SCGs, but the contents of caffeoylquinic acids (i.e., 5-CQA, 4-CQA, 5-CQA, 3,4-diCQA, 3,5-diCQA, and 4,5-diCQA) in Arabica SCGs were higher than that in Robusta SCGs (Bravo et al., 2012). Furthermore, the amounts of bioactive compounds in SCGs have been previously documented to be highly dependent on extraction techniques and solvents. Many extraction methods (i.e., solid-liquid extraction, supercritical fluid extraction, Soxhlet extraction, and ultrasound, and microwave) with different solvents i.e., polar (methanol and ethanol), medium or non-polar (e.g., dichloromethane, ethyl acetate, hexane, supercritical fluids, subcritical water, deep eutectic and supramolecular solvents) have been utilized to maximize the recovery of bioactive compounds in SCGs (Mussatto et al., 2011; Andrade et al., 2012; Pavlović et al., 2013; Getachew and Chun, 2017; Yoo et al., 2018; Torres-Valenzuela et al., 2019). Spent coffee grounds defatted with petroleum ether (1:11, w/v) for 3 h at 60°C in a Soxhlet extraction system yielded the highest contents of total phenolic compounds, which ranged 18–22 mg 3-CQA equivalents/g SCG (Andrade et al., 2012). An increase in roasting levels of coffee beans significantly reduced the total phenolic compounds in SCGs. In fact, total phenolic compounds of SCGs derived from medium roasted coffee beans were 9.9 mg/g, while that of SCGs obtained from dark roasted coffee beans were 4.6 mg/g SCG (López-Barrera et al., 2016). Future efforts are ongoing to identify optimum roasting conditions that maximize the cytokine suppressive activities of the SCG extracts.

## Conclusion

Methanolic extracts of SCGs possessed cytokine inhibition on the human pro-monocytic cell line U-937. The cytokine suppressive effects and the contents of anti-inflammatory compounds in SCGs obtained from different Arabica cultivars were variable. Hawaiian Kona extracts showed the strongest inhibitory effect on the secretion of all 3 cytokines (TNF-α, IL-6, and IL-10), while Ethiopian Yirgacheffe extract reduced the secretions of TNF-α and IL-6 and Costa Rican Tarrazu only decreased the secretion of IL-6. Multiple (26) metabolites in the SCG extracts with known anti-inflammatory activities were identified via an untargeted metabolomics analysis. Targeted (LC-MS/MS) analyses resulted in the quantification of 12 metabolites that had high relative intensities in all of the extracts. Spent coffee grounds contain a wealth of anti-inflammatory bioactive compounds with caffeine and 5-CQA as the most abundant compounds. Our findings indicate that SCGs could be promising sources of anti-inflammatory bioactive compounds that can be utilized for pharmaceutical and cosmetic industries.

## Data Availability Statement

The datasets generated for this study are available on request to the corresponding author.

## Author Contributions

C-HL and K-VH contributed to conception of the study. K-VH wrote the first draft of the manuscript. K-VH, KS, and C-HL designed the experiments. K-VH, KS, JP, and PV performed the experiments. K-VH and KS performed the analyses. KS, ZL, LS, and C-HL provided materials required for the experiments. All authors edited and approved the final version of the manuscript.

## Conflict of Interest

The authors declare that the research was conducted in the absence of any commercial or financial relationships that could be construed as a potential conflict of interest.

## Footnotes

1 www.xcmsonline.scripps.edu/

2 http://metlin.scripps.edu
