# Supplementary material for: Identification and Quantification of Bioactive Molecules Inhibiting Pro-inflammatory Cytokine Production in Spent Coffee Grounds Using Metabolomics Analyses
Source: Front Pharmacol. 2020 Mar 6;11:229. doi: 10.3389/fphar.2020.00229 (PMC7073796; doi:10.3389/fphar.2020.00229)
Supplement: Supplementary file 1 [file Table_1.docx]

**Supplementary Information**

**Identification and Quantification of Bioactive Molecules Inhibiting Pro-inflammatory Cytokine Production in Spent Coffee Grounds Using Metabolomics Analyses**

Khanh-Van Ho^1,2^, Kathy L. Schreiber^3^, Jihyun Park^1^, Phuc Vo^1^, Zhentian Lei^4,5^, Lloyd W. Sumner^4,5^, Charles R. Brown^6^, and Chung-Ho Lin^1*^

^1^Center for Agroforestry, School of Natural Resources, University of Missouri, Columbia, Missouri, USA

^2^Department of Food Technology, Can Tho University, Can Tho, Vietnam

^3^Cell and Immunobiology Core, University of Missouri, Columbia, Missouri, USA

^4^Metabolomics Center, University of Missouri, Columbia, Missouri, USA

^5^Department of Biochemistry, Bond Life Sciences Center, University of Missouri, Columbia, Missouri, USA

^6^Department of Veterinary Pathobiology, University of Missouri, Columbia, Missouri, USA

***Correspondence:**Corresponding Author:
Chung-Ho Lin: LinChu@missouri.edu

**Supplementary Table 1**. Putative metabolites with known to produce anti-inflammatory activities in the methanolic extracts of spent coffee grounds identified via an untargeted metabolomics approach

| **No.** | **Putatively identified compound** | **Anti-inflammatory activity** |
| --- | --- | --- |
| 1 | 3-Caffeoylquinic acid | Hwang et al. (2014) |
| 2 | 5-Caffeoylquinic acid | Kim et al. (2015) |
| 3 | 3,5-Caffeoylquinic acid | Hong et al. (2015) |
| 4 | Caffeic acid | Chao et al. (2009) |
| 5 | Caffeine | Köroğlu et al. (2014) |
| 6 | Catechin | Nakanishi et al. (2010) |
| 7 | Chrysin | Ahad et al. (2014) |
| 8 | Daidzein | Liu et al. (2009) |
| 9 | Epicatechin | Morrison et al. (2014) |
| 10 | Eugenol | Yogalakshmi et al. (2010) |
| 11 | Ferulic acid | Zhu et al. (2014) |
| 12 | Gallic acid | Kim et al. (2005) |
| 13 | Naringenin | Pinho-Ribeiro et al. (2016) |
| 14 | Naringin | Sahu et al. (2014) |
| 15 | Oxyresveratrol | Wei et al. (2017) |
| 16 | p-Coumaric acid | Pragasam et al. (2013) |
| 17 | p-Hydroxybenzoic acid | Manuja et al. (2013) |
| 18 | Pectolinarin | Lim et al. (2008) |
| 19 | Quercetin | Guardia et al. (2001) |
| 20 | Quinic acid | Åkesson et al. (2005) |
| 21 | Resveratrol | Nunes et al. (2018) |
| 22 | Rutin | Guardia et al. (2001) |
| 23 | Tectochrysin | Hou et al. (2018) |
| 24 | Theaflavin | Zu et al. (2012) |
| 25 | Vanillic acid | Kim et al. (2011) |
| 26 | Vitexin rhamnoside | Tadić et al. (2008) |

**References**

Ahad, A., A. A. Ganai, M. Mujeeb, and W. A. Siddiqui. 2014. Chrysin, an anti-inflammatory molecule, abrogates renal dysfunction in type 2 diabetic rats. Toxicol. Appl. Pharmacol 279: 1-7.

Åkesson, C., H. Lindgren, R. W. Pero, T. Leanderson, and F. Ivars. 2005. Quinic acid is a biologically active component of the Uncaria tomentosa extract C-Med 100®. Int. Immunopharmacol. 5: 219-229.

Chao, P.-c., C.-c. Hsu, and M.-c. Yin. 2009. Anti-inflammatory and anti-coagulatory activities of caffeic acid and ellagic acid in cardiac tissue of diabetic mice. Nutr. Metab. 6: 33.

Guardia, T., A. E. Rotelli, A. O. Juarez, and L. E. Pelzer. 2001. Anti-inflammatory properties of plant flavonoids. Effects of rutin, quercetin and hesperidin on adjuvant arthritis in rat. Il farmaco 56: 683-687.

Hong, S., T. Joo, and J.-W. Jhoo. 2015. Antioxidant and anti-inflammatory activities of 3, 5-dicaffeoylquinic acid isolated from *Ligularia fischeri* leaves. Food Sci. Biotechnol. 24: 257-263.

Hou, R., Y. Han, Q. Fei, Y. Gao, R. Qi, R. Cai, and Y. Qi. 2018. Dietary Flavone Tectochrysin exerts anti‐inflammatory action by directly inhibiting MEK1/2 in LPS‐primed macrophages. Mol. Nutr. Food Res. 62: 1700288.

Hwang, S. J., Y.-W. Kim, Y. Park, H.-J. Lee, and K.-W. Kim. 2014. Anti-inflammatory effects of chlorogenic acid in lipopolysaccharide-stimulated RAW 264.7 cells. Inflamm. Res. 63: 81-90.

Kim, M.-C., S.-J. Kim, D.-S. Kim, Y.-D. Jeon, S. J. Park, H. S. Lee, J.-Y. Um, and S.-H. Hong. 2011. Vanillic acid inhibits inflammatory mediators by suppressing NF-κB in lipopolysaccharide-stimulated mouse peritoneal macrophages. ‎Immunopharmacol. Immunotoxicol. 33: 525-532.

Kim, M., S.-Y. Choi, P. Lee, and J. Hur. 2015. Neochlorogenic acid inhibits lipopolysaccharide-induced activation and pro-inflammatory responses in BV2 microglial cells. Neurochem. Res. 40: 1792-1798.

Kim, S.-H., C.-D. Jun, K. Suk, B.-J. Choi, H. Lim, S. Park, S. H. Lee, H.-Y. Shin, D.-K. Kim, and T.-Y. Shin. 2005. Gallic acid inhibits histamine release and pro-inflammatory cytokine production in mast cells. Toxicol. Sci. 91: 123-131.

Köroğlu, Ö. A., P. M. MacFarlane, K. V. Balan, W. J. Zenebe, A. Jafri, R. J. Martin, and P. Kc. 2014. Anti-inflammatory effect of caffeine is associated with improved lung function after lipopolysaccharide-induced amnionitis. Neonatology 106: 235-240.

Lim, H., K. H. Son, H. W. Chang, K. Bae, S. S. Kang, and H. P. Kim. 2008. Anti-inflammatory activity of pectolinarigenin and pectolinarin isolated from *Cirsium chanroenicum*. Biol. Pharm. Bull. 31: 2063-2067.

Liu, M.-H., Y.-S. Lin, S.-Y. Sheu, and J.-S. Sun. 2009. Anti-inflammatory effects of daidzein on primary astroglial cell culture. Nutr. Neurosci. 12: 123-134.

Manuja, R., S. Sachdeva, A. Jain, and J. Chaudhary. 2013. A comprehensive review on biological activities of p-hydroxy benzoic acid and its derivatives. Int. J. Pharm. Sci. Rev. Res 22: 109-115.

Morrison, M., R. van der Heijden, P. Heeringa, E. Kaijzel, L. Verschuren, R. Blomhoff, T. Kooistra, and R. Kleemann. 2014. Epicatechin attenuates atherosclerosis and exerts anti-inflammatory effects on diet-induced human-CRP and NFκB in vivo. Atherosclerosis 233: 149-156.

Nakanishi, T., K. Mukai, H. Yumoto, K. Hirao, Y. Hosokawa, and T. Matsuo. 2010. Anti‐inflammatory effect of catechin on cultured human dental pulp cells affected by bacteria‐derived factors. Eur. J. Oral Sci. 118: 145-150.

Nunes, S., F. Danesi, D. Del Rio, and P. Silva. 2018. Resveratrol and inflammatory bowel disease: The evidence so far. Nutr. Res. Rev. 31: 85-97.

Pinho-Ribeiro, F. A., A. C. Zarpelon, V. Fattori, M. F. Manchope, S. S. Mizokami, R. Casagrande, and W. A. Verri Jr. 2016. Naringenin reduces inflammatory pain in mice. Neuropharmacology 105: 508-519.

Pragasam, S. J., V. Venkatesan, and M. Rasool. 2013. Immunomodulatory and anti-inflammatory effect of p-coumaric acid, a common dietary polyphenol on experimental inflammation in rats. Inflammation 36: 169-176.

Sahu, B. D., S. Tatireddy, M. Koneru, R. M. Borkar, J. M. Kumar, M. Kuncha, R. Srinivas, and R. Sistla. 2014. Naringin ameliorates gentamicin-induced nephrotoxicity and associated mitochondrial dysfunction, apoptosis and inflammation in rats: possible mechanism of nephroprotection. Toxicol. Appl. Pharmacol. 277: 8-20.

Tadić, V. M., S. Dobrić, G. M. Marković, S. M. Ðorđević, I. A. Arsić, N. a. R. Menković, and T. Stević. 2008. Anti-inflammatory, gastroprotective, free-radical-scavenging, and antimicrobial activities of hawthorn berries ethanol extract. J. Agric. Food Chem. 56: 7700-7709.

Wei, J., J.-R. Chen, E. M. A. Pais, T.-Y. Wang, L. Miao, L. Li, L.-Y. Li, F. Qiu, L.-M. Hu, and X.-M. Gao. 2017. Oxyresveratrol is a phytoestrogen exerting anti-inflammatory effects through NF-κB and estrogen receptor signaling. Inflammation 40: 1285-1296.

Yogalakshmi, B., P. Viswanathan, and C. V. Anuradha. 2010. Investigation of antioxidant, anti-inflammatory and DNA-protective properties of eugenol in thioacetamide-induced liver injury in rats. Toxicology 268: 204-212.

Zhu, H., Q.-H. Liang, X.-G. Xiong, J. Chen, D. Wu, Y. Wang, B. Yang, Y. Zhang, Y. Zhang, and X. Huang. 2014. Anti-inflammatory effects of the bioactive compound ferulic acid contained in oldenlandia diffusa on collagen-induced arthritis in rats. Evid.-Based Complementary Altern. Med. 2014.

Zu, M., F. Yang, W. Zhou, A. Liu, G. Du, and L. Zheng. 2012. In vitro anti-influenza virus and anti-inflammatory activities of theaflavin derivatives. Antivir. Res. 94: 217-224.
